# Supplementary material for: Developmentally Programmed Switches in DNA Replication: Gene Amplification and Genome-Wide Endoreplication in Tetrahymena
Source: Microorganisms. 2023 Feb 16;11(2):491. doi: 10.3390/microorganisms11020491 (PMC9967165; doi:10.3390/microorganisms11020491)
Supplement: Supplementary file 1 [file microorganisms-11-00491-s001.zip › microorganisms-2121092-supplementary.pdf]

microorganisms

MDPI

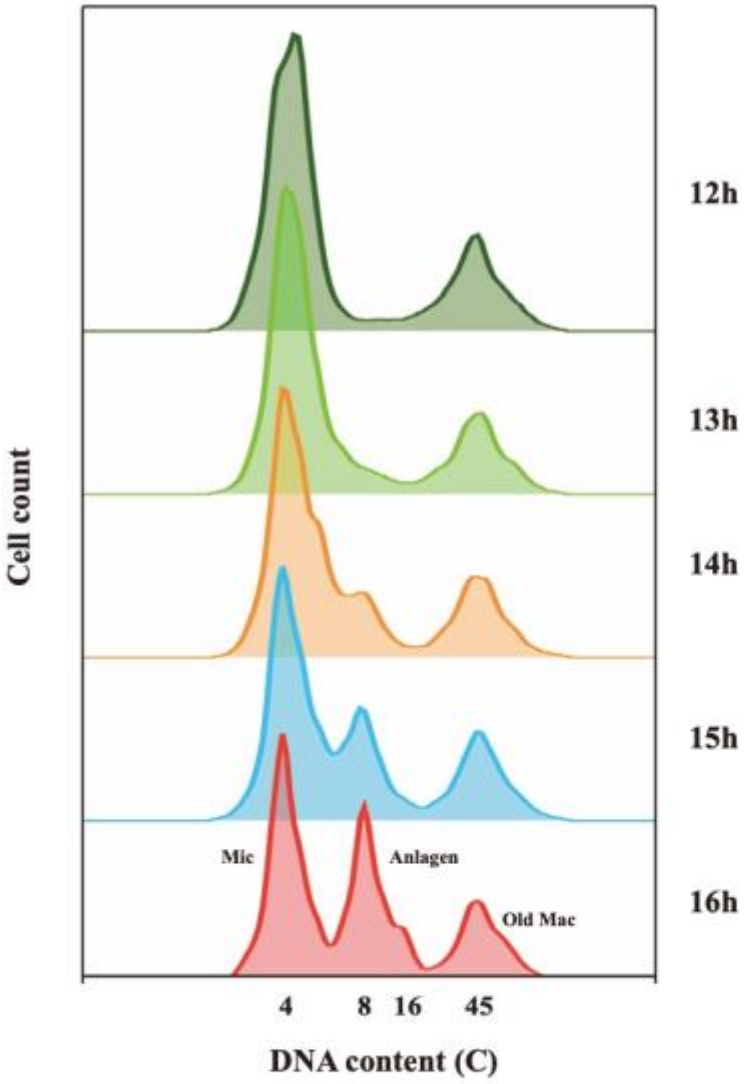

Supplemental Figure S1. Flow cytometry profile at 1h intervals during Endoreplication Phase 1 (cross: SB1943 x SB 4204).

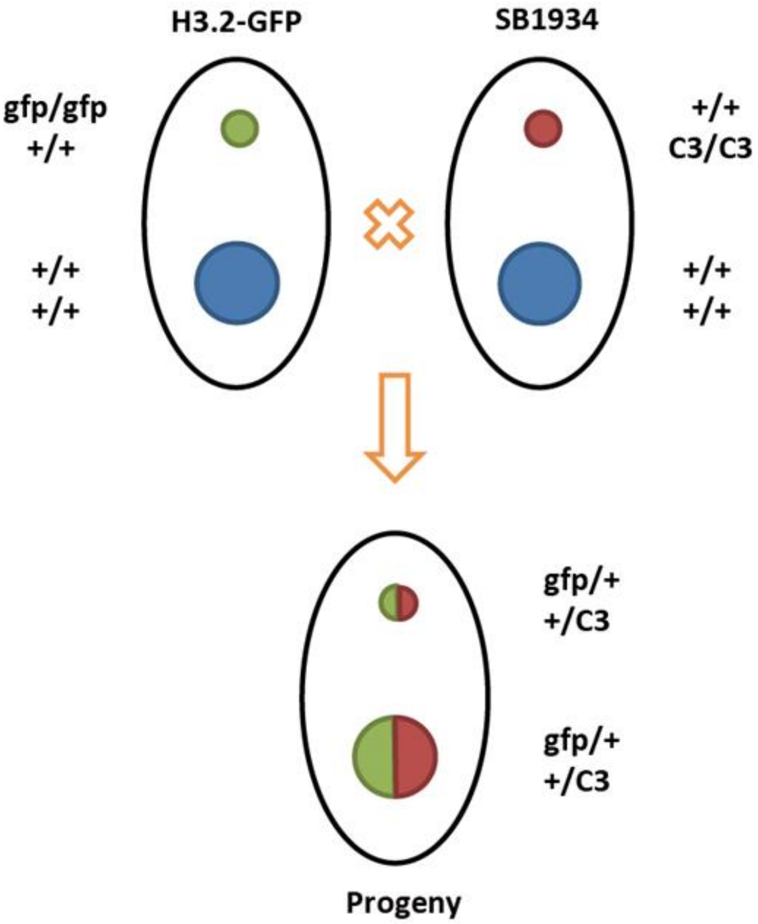

Supplemental Figure S2. Schematic of mating between the two heterokaryons strains SB1934 (homozygous C3 rDNA micronucleus, B rDNA macronucleus) and H3.2-GFP (homozygous H3.2-GFP tagged micronucleus, wild type H3.2 macronucleus).
